# Multidimensional predictors of psychological distress in dental education: the mediating role of academic stress a structural equation modelling study with competing model specification

**DOI:** 10.1186/s12909-026-09957-3

**Published:** 2026-07-31

**Authors:** Mehmet Erşan Kalaycı, Abdulrahman Neccaroğlu, Elsu Yaşar, Davut Çelik

**Affiliations:** 1 Basic Medical Sciences, Department of Biostatistics and Medical Informatics, Faculty of Medicine, Karadeniz Technical University, Trabzon, Türkiye Turkey; 2 Department of Endodontics, Faculty of Dentistry, Karadeniz Technical University, Trabzon, Türkiye Turkey; 3 Faculty of Dentistry, Karadeniz Technical University, Trabzon, Türkiye Turkey

**Keywords:** Dental students, Academic stress, Anxiety, Depression, Psychological distress, Structural equation modelling, GAD-7, Mediation

## Abstract

**Background:**

Dental students experience disproportionately high levels of psychological distress, with prevalence rates of clinically significant stress ranging from 22 to 50% across international samples. Academic stress has been identified as the primary mediating pathway through which sociodemographic and lifestyle factors influence mental health outcomes. However, it remains unclear whether psychological distress in dental students is better represented by a transdiagnostic mental distress construct or by disorder-specific measures such as GAD-7 within structural equation modelling frameworks. This study aimed to test a structural equation model (SEM) in which sociodemographic and lifestyle factors predict psychological distress through the mediating role of academic stress, and to compare a transdiagnostic Mental Distress model (Model 1) with a GAD-7 anxiety-specific model (Model 2).

**Methods:**

A cross-sectional survey was administered to 303 dental students (years 1–5) in a Turkish dental school. Instruments included the Academic Stress Scale (ASÖ; 5 items), the GAD-7, and the DASS-21 (Depression and Anxiety subscales). Structural equation modelling was conducted in AMOS 24.0 with 5,000 bootstrap iterations and bias-corrected 95% confidence intervals.

**Results:**

Both models demonstrated acceptable fit. Academic stress fully mediated the relationship between all examined predictors and psychological distress outcomes. Economic status emerged as the strongest predictor of academic stress, followed by gender and year of study. Poor sleep quality was associated with elevated academic stress. The competing model incorporating GAD-7 as an additional latent construct showed comparable fit, confirming the robustness of the mediation structure.

**Conclusions:**

Academic stress acts as a central full mediator between modifiable and sociodemographic factors and psychological distress in dental students. The transdiagnostic model offers superior theoretical fit, while the GAD-7 model provides practical clinical utility for early screening. Interventions targeting economic support, sleep hygiene, and routine GAD-7 screening are recommended.

**Supplementary Information:**

The online version contains supplementary material available at https://doi.org/10.1186/s12909-026-09957-3.

## Introduction

Mental health difficulties among dental students have attracted increasing research attention over the past two decades. Systematic reviews have consistently documented that dental students experience higher levels of stress, anxiety, and depression compared with both the general student population and the public [1, 2]. Elani et al. [3] synthesized 26 studies and reported that 22–50% of dental students across diverse settings met threshold criteria for clinically significant stress. Similarly, the landmark multi-country survey by Divaris et al. [4] found that academic demands, patient-related concerns, and financial pressures constituted the dominant stressors across dental curricula worldwide. Despite the robustness of these findings, the mechanisms through which such stressors translate into psychological distress remain insufficiently understood.

### Psychological distress in dental education

Dental education is consistently identified as one of the most psychologically demanding training environments in healthcare. Studies conducted across multiple countries report that between 40% and 80% of dental students experience clinically significant levels of stress, anxiety, or depression during their training [3, 5, 6]. The sources of distress are multifactorial, encompassing academic workload, clinical performance demands, financial burden, interpersonal conflicts, and uncertainty about professional competence [7, 8]. Comparative evidence suggests that dental students report higher levels of psychological distress than medical students and age-matched general population samples [9, 10]. For instance, Acharya [11] and colleagues reported that over 70% of dental students in India experienced “high levels of stress.” In a global meta-analysis, the prevalence of burnout syndrome among dental students was found to be 26.5% [12]. Similarly, cross-sectional studies conducted in different countries have reported anxiety levels ranging between 40% and 60% among students [6, 13]. For example, Basudan et al. [6] in Saudi Arabia reported that approximately half of the participants had moderate to severe levels of anxiety and depression. In Turkey, some studies have shown that 30–50% of dental students experience intense anxiety before clinical examinations [14, 15].

Anxiety and depression are among the most reported mental health problems in dental student populations across multiple countries [3, 9, 16], though prevalence estimates vary considerably by region, measurement instrument, and sampling strategy. Importantly, these two conditions are closely related: anxiety frequently precedes and mediates the development of depressive symptoms, in line with the stress-generation model and the tripartite model of anxiety and depression [17, 18]. The GAD-7 scale [19] has become a widely used, brief, and psychometrically robust tool for assessing generalized anxiety in both clinical and research settings.

### Key factors influencing psychological distress

Academic stress broadly defined as a student’s perception of the demands associated with academic life as exceeding available coping resources [20] has been consistently identified as the primary mediating variable in the relationship between contextual pressures and psychological outcomes. Alzahem et al. [16] identified curriculum overload, patient management, and performance anxiety as the principal academic stressors specific to dental education. Subsequent work has emphasized that the transition from preclinical to clinical training (typically occurring in the third and fourth years) represents a particularly vulnerable period, characterized by a sudden increase in direct patient responsibility and complex technical demands [21].

Beyond the academic environment itself, a constellation of sociodemographic and lifestyle factors has been linked to elevated distress in dental and health-professional student populations [3, 16, 22]. Economic hardship consistently emerges as a potent predictor: the high material costs of dental education including tuition fees, clinical equipment, and laboratory supplies place particular financial strain on students [21, 22]. For example, many students experience economic concerns while covering their educational expenses through family support or loans, which further increases overall stress levels [23]. Gender differences in psychological distress are well-established, with female dental students typically reporting higher levels of perceived stress and anxiety than their male counterparts [6, 24], a pattern attributed to differential socialization, role strain, and biological vulnerability factors. Sleep quality and duration represent a modifiable lifestyle variable that bidirectionally interacts with psychological distress: insufficient sleep impairs emotional regulation and cognitive performance, thereby amplifying the subjective burden of academic demands [25, 26].

More specific findings at the risk factor level have also been reported. Studies generally show that students in the early stages of clinical education (third- or fourth-year students) experience the highest levels of stress and anxiety [10, 27]. During clinical training, students often experience anxiety related to the fear of failure or causing pain to patients, which is a significant contributing factor to increased anxiety levels [28]. This is attributed to clinical practice pressure and anxiety stemming from inexperience in patient management. Social factors also play an important role; for example, parental expectations, a competitive peer environment, and insufficient social support can negatively affect students’ psychological well-being [6, 29]. Research also indicates that personality traits and coping mechanisms are influential. Perfectionistic tendencies, high self-criticism, and low self-esteem reduce the ability to cope with stress [30, 31]. Overall, traits such as high perfectionism and sensitivity among dental students, when combined with a highly demanding academic environment, increase the risk of psychological disorders [32]. All of these risk factors increase psychological vulnerability in the demanding environment faced by dental students; consequently, both the causes and manifestations of stress vary considerably among students [6, 32].

Importantly, these factors may not exert direct independent effects on psychological distress. Rather, they are likely to operate indirectly by increasing perceived academic stress, suggesting that academic stress functions as a central mediating pathway linking diverse risk factors to mental health outcomes. Recent studies have begun to test such mediation frameworks. Shekarchizadeh et al. [33] demonstrated that dental environmental stress mediated the relationship between learning environment quality and academic burnout among 300 dental students using structural equation modelling. Similarly, emotion regulation strategies have been shown to partially mediate the stress-burnout pathway in dental postgraduate students [34]. Despite these emerging efforts, the specific role of academic stress as a mediator between sociodemographic factors and transdiagnostic psychological distress has rarely been formally tested using comprehensive multivariate approaches.

### The importance of reducing psychological distress

Psychological distress among dental students carries serious consequences beyond academic performance. The negative consequences of high levels of stress on students have also been extensively examined in the literature. Elevated stress levels have been associated with compromised patient care quality, increased risk of professional burnout, and higher attrition rates [35, 36]. Long-term stress and anxiety may develop into depression and burnout, posing a serious threat to students’ mental health [6, 37]. For example, studies have found significantly higher prevalence rates of burnout syndrome and depression among dental students experiencing chronic stress [37]. In some countries, even increased rates of suicide attempts or serious psychiatric admissions have been reported among dental students [37, 38].

In social life, stressed students may experience withdrawal from family and peer relationships and a decrease in motivation, which can lead to disengagement from their education [10]. Students who experience chronic stress during their education are more likely to develop burnout, professional dissatisfaction, and a higher tendency to make clinical errors later in their careers [1, 3, 39]. This situation threatens not only the individual well-being of students but also the quality of oral health services provided to society [40, 41].

Early identification and intervention are therefore essential not only for student well-being but also for the quality and safety of clinical training. Supporting dental students both academically and personally throughout their education is critical for their future professional success and the quality of patient care [1, 3].

Institutional-level strategies, including mental health support services, curriculum redesign, financial aid programs, and peer support networks, have demonstrated effectiveness in reducing distress levels in dental student populations [16, 42]. In addition, developing mental health support programs at the university level, implementing systematic screening, and improving counseling services would both reduce student attrition and positively contribute to long-term professional development [15, 43]. In conclusion, given the prevalence and consequences of psychological disorders among dental students, this issue represents both a public health concern and an important matter in educational management.

Despite these efforts, the effectiveness of such interventions remains inconsistent. One potential explanation is the lack of a clearly defined mechanism linking risk factors to psychological outcomes. Without such a framework, interventions may fail to target the most influential processes underlying distress. Identifying whether academic stress constitutes a central mediating mechanism is therefore essential for developing more targeted and effective strategies.

### Methodological gaps and study aims

Despite the richness of this descriptive literature, several methodological gaps remain. First, most studies have employed single-mediator models or simple bivariate analyses, failing to capture the indirect pathways through which distal predictors exert their effects on mental health outcomes [33]. Structural equation modelling (SEM) offers a powerful framework for simultaneously estimating measurement models, direct effects, and indirect (mediation) pathways within a single analysis [44–46]. Second, the choice of mediating construct matters theoretically: a transdiagnostic formulation (operationalized through a latent Mental Distress factor) allows broader inference, while an anxiety-specific operationalization (such as the GAD-7) more directly aligns with clinical screening practice. To our knowledge, no study has formally compared these two competing SEM specifications in the dental student literature.

The present study therefore aimed (i) to test a full SEM with economic difficulties, gender, academic year and sleep duration as predictors of academic stress (ASÖ), and academic stress as predictor of a latent psychological distress outcome operationalized via DASS-21 Depression and Anxiety subscales; (ii) to compare a transdiagnostic Mental Distress mediator specification (Model 1) with an alternative GAD-7-based specification (Model 2); and (iii) to quantify bootstrap-based indirect effects for all predictor variables in both specifications.

## Methods

### Study design and participants

A cross-sectional survey design was employed. All registered dental students (years 1–5) at a Turkish dental school were invited to participate during the 2025–2026 academic year. A total of 303 students returned complete and usable questionnaires. Ethical approval was granted by the institutional review board prior to data collection. Written informed consent was obtained from all participants. The a priori power analysis revealed that a minimum sample size of 244 participants would be sufficient to detect a medium effect size (f² = 0.05) with 80% power at α = 0.05. The current sample (*N* = 303) surpasses this requirement, thereby providing adequate statistical power for the regression analyses. Prior to analysis, data validity was verified through examination of missing data patterns, identification and assessment of univariate and multivariate outliers, and confirmation of distributional assumptions; no cases were excluded on these grounds beyond the initial completeness screening.

### Measures

Sociodemographic and lifestyle variables were assessed by self-report (Supplementary Table S1). Economic difficulties were rated on a five-point scale (1 = no difficulty; 5 = severe difficulty). Academic year was treated as a continuous variable (1–5), with years 1–3 representing preclinical training and years 4–5 clinical training. Sleep duration was categorized as: 1 = < 4 h; 2 = 4–6 h; 3 = 6–8 h; 4 = > 8 h. Gender was dummy-coded (0 = male; 1 = female). These items were adapted from commonly used demographic questions discussed in the educational research literature [16].

Academic stress was measured using the five highest-loading items (1, 2, 3, 7, and 8) from the Turkish version of the Academic Stress Scale (ASÖ; Supplementary Table S2) [47]. The subscale demonstrated good internal consistency in the present sample (Cronbach’s α = 0.866). Corrected item-total correlations ranged from 0.623 to 0.725, confirming the reliability of the selected items.

The Generalized Anxiety Disorder 7-item scale (GAD-7; Supplementary Table S3) [19] was used as an observed anxiety mediator in Model 2. The GAD-7 assesses the frequency of seven core generalized anxiety symptoms over the preceding two weeks. It is one of the most widely validated anxiety screening instruments in clinical and student populations, with cut-offs for mild (≥ 5), moderate (≥ 10), and severe (≥ 15) anxiety established across multiple cultures. Six items (GAD_7_1, 2, 3, 4, 5, 7) were included in the analysis. The subscale demonstrated excellent internal consistency (Cronbach’s α = 0.909), with corrected item-total correlations ranging from 0.693 to 0.817.

Psychological distress outcomes were measured with the Depression (7 items; Supplementary Table S4) and Anxiety (5 items: items 3–7; Supplementary Table S5) subscales of the Turkish DASS-21 [48]. DASS-21 Depression subscale (7 items) demonstrated strong internal consistency (Cronbach’s α = 0.896), with corrected item-total correlations between 0.568 and 0.779. DASS-21 Anxiety subscale (5 items; items 3–7) also showed good reliability (Cronbach’s α = 0.848), with corrected item-total correlations ranging from 0.586 to 0.723.

**Fig. 1:**
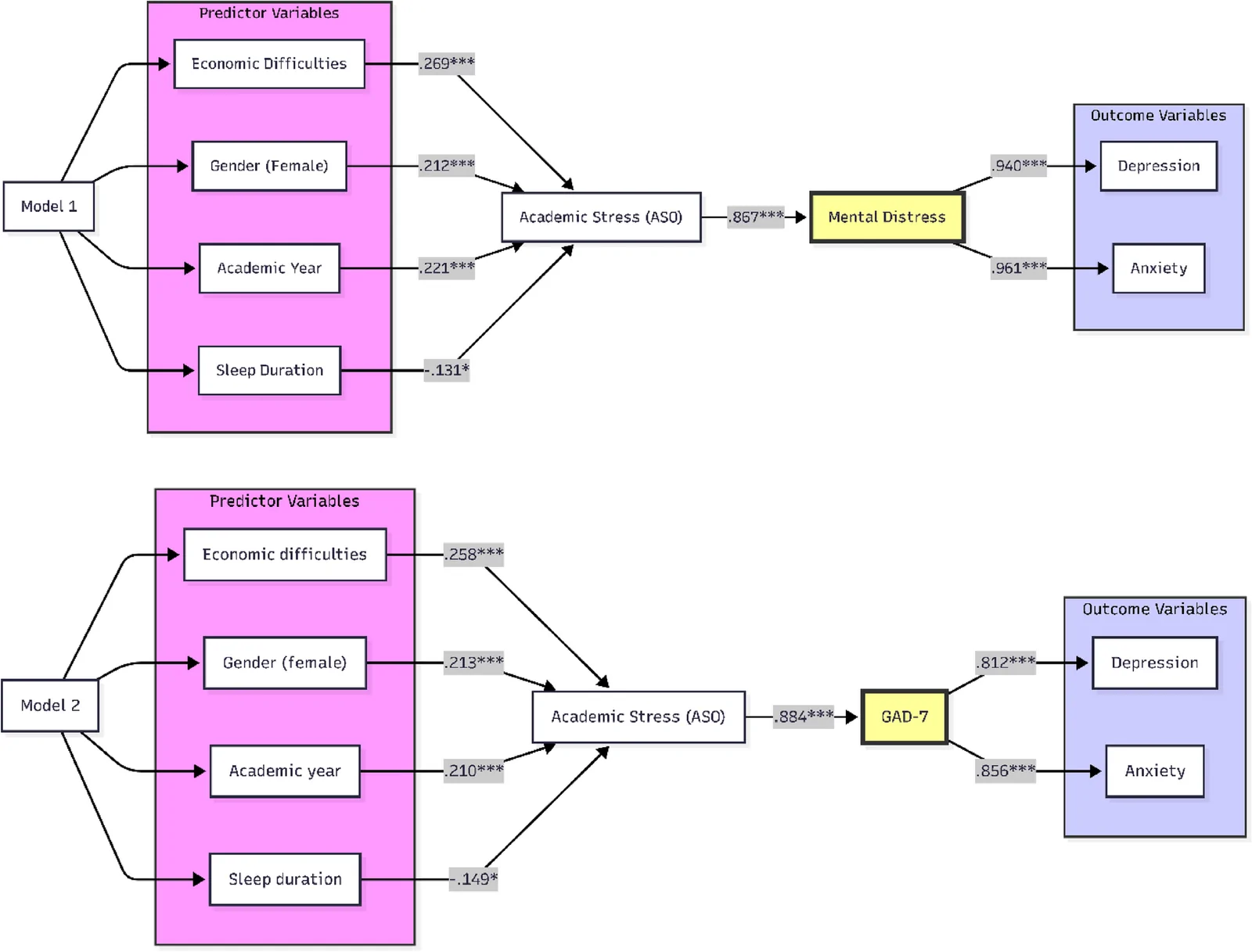
Structural Equation Models with Standardized Path Coefficients. Standardized path coefficients (β) are shown for each model. Model 1 presents a transdiagnostic model with a latent Mental Distress factor mediating the relationship between predictors (economic difficulties, gender, academic year, sleep duration) and outcomes (Depression, Anxiety). Model 2 presents an alternative model where GAD-7 serves as the latent mediator. *p < .05, **p < .01, ***p < .001

Prior to structural model estimation, measurement properties of all latent constructs were evaluated. Convergent validity was assessed using Average Variance Extracted (AVE) and Composite Reliability (CR), and all constructs demonstrated satisfactory internal consistency and convergent validity. In Model 1, AVE values were 0.551 for academic stress (ASÖ), 0.564 for DASS-21 Depression, and 0.502 for DASS-21 Anxiety, with corresponding CR values of 0.859, 0.900, and 0.833. In Model 2, AVE and CR values were very similar for ASÖ (AVE = 0.544, CR = 0.856), Depression (AVE = 0.570, CR = 0.902), and Anxiety (AVE = 0.508, CR = 0.837), while the GAD-7 latent variable demonstrated strong reliability and convergent validity (AVE = 0.624, CR = 0.908). All AVE values exceeded 0.50 and all CR values exceeded 0.70, indicating adequate construct reliability and convergent validity across both model specifications (Fig. 1).

### Statistical analysis

Two priori SEM specifications were estimated in AMOS 24.0 [49] using maximum likelihood estimation. In Model 1 (primary/transdiagnostic model), a first-order confirmatory factor analysis (CFA) latent variable labelled Mental Distress was defined by a second-order structure in which DASS-21 Depression and DASS-21 Anxiety each served as latent factors predicting the overarching distress construct. In Model 2 (alternative/anxiety-specific model), the GAD-7 items constituted a first-order latent variable that directly mediated the path from academic stress to DASS-21 outcomes.

Both models shared identical exogenous predictors (economic difficulties, gender, academic year, sleep duration) and the same ASÖ measurement model. Model fit was evaluated using the following indices and established benchmarks [50, 51]; χ²/df < 3.0; CFI ≥ 0.90; TLI ≥ 0.90; RMSEA ≤ 0.08 (with 90% CI); SRMR ≤ 0.08. The Akaike Information Criterion (AIC) and Bayesian Information Criterion (BIC) were used for comparative model selection; lower values indicate better parsimony-adjusted fit.

Indirect (mediation) effects were estimated using bias-corrected bootstrap confidence intervals based on 5,000 resampled datasets. An effect was considered statistically significant if the 95% bootstrap CI excluded zero. All standardized regression weights (β) and their 95% CIs are reported. Due to the cross-sectional design, the term ‘predictor’ rather than ‘cause’ is used throughout. Data screening confirmed acceptable normality and no extreme multicollinearity.

### Data analysis

There are two structural equation modeling approaches in the literature with different underlying logic: covariance-based SEM (CB-SEM) and variance-based SEM (PLS-SEM). CB-SEM (AMOS) is a covariance-based structural equation modelling approach used to test and confirm existing theories by evaluating how well the hypothesized model reproduces the observed covariance matrix, assessed through global fit indices such as CFI, RMSEA, and SRMR.PLS-SEM (SmartPLS) is a variance-based structural equation modelling approach focused on maximizing the explained variance (R²) in dependent constructs, making it particularly suitable for prediction-oriented research and theory development, especially when sample sizes are limited or theoretical foundations are less established [52, 53].

Although Partial Least Squares Structural Equation Modeling (PLS-SEM) offers advantages in estimator research and in cases where distributional assumptions are violated [54, 55], the confirmatory nature of this study, the use of well-established measurement scales with strong psychometric properties, and the aim of testing and validating existing theory led to the preference for Covariance-Based Structural Equation Modeling (CB-SEM). Data was analyzed using Covariance-Based Structural Equation Modelling (CB-SEM) implemented in IBM AMOS 24 [49]. CB-SEM was selected over Partial Least Squares SEM (PLS-SEM) for several methodological and theoretical reasons. First, the study adopts a confirmatory research design with clearly specified competing theoretical models and aims to evaluate overall model fit using established global fit indices, including the Chi-square/degrees of freedom ratio (χ²/df ≤ 3.0), Root Mean Square Error of Approximation (RMSEA ≤ 0.08), Comparative Fit Index (CFI ≥ 0.90), and Tucker–Lewis Index (TLI ≥ 0.90), and Standardized Root Mean Square Residual (SRMR ≤ 0,08). Second, the sample size (*N* = 303) exceeds the recommended minimum thresholds for CB-SEM with complex models (typically 200–250 observations), thereby ensuring adequate statistical power for maximum likelihood estimation. Third, data screening procedures confirmed that the dataset satisfied multivariate normality assumptions following appropriate diagnostic tests. Finally, CB-SEM enables more precise parameter estimation and allows robust comparison of both nested and non-nested models using information criteria such as Akaike Information Criterion (AIC) and Bayesian Information Criterion (BIC), which was essential for the competing-model framework employed in this study [53, 56].

Overall, CB-SEM is particularly appropriate when (a) the researcher seeks to test a theoretically specified model against empirical data, (b) the sample size is sufficient for maximum likelihood estimation, and (c) constructs are operationalized using reflective measurement models [46, 57].

Measurement model validity was assessed through confirmatory factor analysis (CFA), with construct reliability evaluated using Composite Reliability (CR > 0.80) and Average Variance Extracted (AVE > 0.50) [53], while discriminant validity was confirmed via Heterotrait-Monotrait Ratio (HTMT) analysis with all values below the 0.85 threshold [58, 59].

Indirect (mediation) effects were tested with bias-corrected bootstrap confidence intervals (5,000 resamples). An effect was considered significant if the 95% CI did not include zero. All analyses used maximum likelihood estimation. Data screening confirmed acceptable normality, no extreme multicollinearity (VIF < 5), and missing data < 5%.

## Results

### Sample characteristics

The final analytic sample comprised 303 dental students (female: *n* = 191, 63%; male: *n* = 112, 37%) drawn from years 1 through 5. Mean economic difficulty score was 2.83(SD = 1.23). A total of 40.9% of participants reported sleeping fewer than 6 h per night. The preclinical-clinical transition group (year 3 and year 4) accounted for 53.8% of respondents.

### Measurement models

All factor loadings in both models were statistically significant at *p* < .001 (5,000 bootstrap iterations, bias-corrected 95% CIs excluding zero). Table 1 presents the standardized factor loadings. For the ASÖ latent construct, loadings ranged from β = 0.661 (ASÖ_7) to β = 0.779 (ASÖ_8) in Model 1, confirming adequate indicator reliability. DASS-21 Depression loadings ranged from β = 0.652 to β = 0.829; DASS-21 Anxiety loadings from β = 0.622 to β = 0.810, across both models. The GAD-7 in Model 2 showed strong loadings (β = 0.723 to β = 0.859), with the highest loading on GAD_7_4 (β = 0.859).

**Table 1:** Standardized Factor Loadings Across Both Model Specifications

Indicator	Factor / Construct	β (Model 1)	β (Model 2)	95% BC CI (M1)
ASÖ_1	ASÖ	0.751	0.727	[0.679, 0.809]
ASÖ_2	ASÖ	0.737	0.724	[0.656, 0.801]
ASÖ_3	ASÖ	0.777	0.815	[0.709, 0.831]
ASÖ_7	ASÖ	0.661	0.642	[0.573, 0.732]
ASÖ_8	ASÖ	0.779	0.768	[0.707, 0.837]
GAD_7_1–7	GAD-7 (M2 only)	—	0.723–0.859	—
DASS_Dep_1–7	Depression	0.652–0.829	0.648–0.835	(range)
DASS_Ank_3–7	Anxiety	0.622–0.810	0.627–0.810	(range)

*BC CI* Bias-corrected bootstrap confidence interval. All loadings are significant at *p* < .001

### Model fit comparison

Table 2 presents the fit statistics for both competing model specifications. Model 1 (transdiagnostic Mental Distress latent variable) demonstrated excellent fit: CMIN/DF = 1.996, CFI = 0.944, TLI = 0.933, RMSEA = 0.057 (90% CI [0.049, 0.066]), SRMR = 0.045, AIC = 461.211, BIC = 665.466. Model 2 (GAD-7 latent mediator; 67 free parameters, df = 311) showed acceptable but marginally inferior fit: CMIN/DF = 2.079, CFI = 0.928, TLI = 0.919, RMSEA = 0.060 (90% CI [0.053, 0.066]), SRMR = 0.054, AIC = 780.526, BIC = 1029.346. On all model-selection indices (CFI, TLI, RMSEA, AIC, BIC), Model 1 was superior. Accordingly, Model 1 was selected as the primary model and Model 2 is presented as the alternative specification.

**Table 2:** Model Fit Indices for the Two Competing SEM Specifications

Fit Index	Model 1 Mental Distress	Model 2 GAD-7	Criterion
CMIN/DF	1.996	2.079	< 3.0
CFI	0.944	0.928	≥ 0.90
TLI (rho2)	0.933	0.919	≥ 0.90
RMSEA	0.057 [0.049, 0.066]	0.060 [0.053, 0.066]	≤ 0.08
GFI	0.896	0.859	≥ 0.90
AGFI	0.864	0.828	≥ 0.80
NFI	0.895	0.872	≥ 0.90
PCLOSE	0.081	0.007	≥ 0.05
χ² (CMIN)	351.211	646.526	—
AIC	461.211	780.526	Lower = better
BIC	665.466	1029.346	Lower = better
SRMR	0.045	0.054	≤ 0.08
df	176	311	
N parameters	55	67	—

*BC CI* bias-corrected bootstrap confidence interval. *CFI* Comparative Fit Index, *TLI* Tucker–Lewis Index, *RMSEA* Root Mean Square Error of Approximation, *GFI* Goodness-of-Fit Index, *AGFI* Adjusted GFI, *NFI* Normed Fit Index; *PCLOSE* probability that RMSEA ≤ 0.05

### Structural paths: direct effects

Table 3 presents standardized regression weights and bootstrap confidence intervals for all structural paths in both models. The pattern of direct effects on ASÖ was consistent across specifications. Economic difficulties was the strongest predictor (Model 1: β = 0.269, *p* < .001 [95% CI 0.149, 0.386]; Model 2: β = 0.258), followed by academic year (Model 1: β = 0.221, *p* < .001 [0.100, 0.332]), gender (Model 1: β = 0.212, *p* < .001 [0.092, 0.318]), and sleep duration as a protective factor (Model 1: β = −0.131, *p* = .021 [− 0.245, − 0.015]). Combined, these predictors explained approximately 20% of the variance in academic stress (R² ≈ 0.20).

Academic stress was a powerful predictor of the latent mediator in both models: β = 0.867 (95% CI [0.808, 0.916]) in Model 1 and β = 0.884 ([0.835, 0.928]) in Model 2, explaining approximately 75% and 78% of variance in the respective mediator constructs. In Model 1, Mental Distress predicted Depression (β = 0.940, 95% CI [0.891, 0.984]) and Anxiety (β = 0.961, 95% CI [0.907, 1.005]). In Model 2, the GAD-7 latent variable predicted Depression (β = 0.812, 95% CI [0.763, 0.856]) and Anxiety (β = 0.856, 95% CI [0.802, 0.902]). Effect sizes for direct paths are reported as standardized regression coefficients (β); following Cohen’s (1988) conventions, β values of 0.10, 0.30, and 0.50 represent small, medium, and large effects, respectively. The strongest predictor, economic difficulties, exhibited a small-to-medium effect (β = 0.269), while the combined model explained a medium proportion of variance in academic stress (R² ≈ 0.20, f² = 0.25, a medium-to-large effect by Cohen’s criteria [60]). The path from academic stress to the mediator yielded a large effect in both models (β = 0.867–0.884, R² ≈ 0.75–0.78).

**Table 3:** Standardized Direct Effects and 95% Bootstrap Confidence Intervals

Structural Path	β M1 (Mental Distress)	β M2 (GAD-7)	*p*	95% BC CI (M1)
Predictors → Academic Stress (ASÖ)				
Economic difficulties → ASÖ	0.269	0.258	< 0.001	[0.149, 0.386]
Gender (female) → ASÖ	0.212	0.213	< 0.001	[0.092, 0.318]
Academic year → ASÖ	0.221	0.210	< 0.001	[0.100, 0.332]
Sleep duration → ASÖ	−0.131	−0.149	< 0.021	[− 0.245, − 0.015]
Academic Stress → Mediator → Outcomes				
ASÖ → Mental Distress / GAD-7	0.867	0.884	< 0.001	[0.808, 0.916]
Mental Distress / GAD-7 → Depression	0.940	0.812	< 0.001	[0.891, 0.984]
Mental Distress / GAD-7 → Anxiety	0.961	0.856	< 0.001	[0.907, 1.005]

### Indirect (Mediation) effects

Bootstrap-based indirect effects (5,000 iterations, bias-corrected) confirmed significant mediation for all predictors in both model specifications (Table 4). In Model 1, economic difficulties exerted the largest standardized indirect effect on Depression (β = 0.219, 95% CI [0.121, 0.321]) and on Anxiety (β = 0.224). Gender (β = 0.173 on Depression; 0.176 on Anxiety), academic year (β = 0.180; 0.184), and sleep (β = −0.107; −0.109) all showed significant indirect effects, with 95% CIs excluding zero. The pattern was highly similar in Model 2: indirect effects on Depression ranged from 0.153 (gender) to 0.185 (economic difficulties), while indirect effects on Anxiety ranged from 0.161 to 0.195. In both specifications, all direct paths from predictors to outcomes were zero (full mediation via academic stress).

**Table 4:** Standardized Indirect Effects via Academic Stress (ASÖ) and Mediator in Both Models

Indirect Effect Path	β M1 → Dep	β M1 → Anx	β M2 → Dep	β M2 → Anx	95% BC CI (M1 → Dep)
Economic diff. → ASÖ → [Med] → Dep	0.219	0.224	0.185	0.195	[0.121, 0.321]
Gender → ASÖ → [Med] → Dep	0.173	0.176	0.153	0.161	[0.078, 0.264]
Academic year → ASÖ → [Med] → Dep	0.180	0.184	0.151	0.159	[0.082, 0.274]
Sleep → ASÖ → [Med] → Dep	−0.107	−0.109	−0.107	−0.113	[− 0.203, − 0.013]

M1 = Model 1 (Mental Distress mediator); M2 = Model 2 (GAD-7 mediator); Dep = DASS-21 Depression; Anx = DASS-21 Anxiety; [Med] = respective mediating construct. All 95% bootstrap CIs exclude zero (*p* ≤ .021). *BC CI* bias-corrected confidence interval

### Discriminant validity: HTMT analysis

As part of construct validity assessment, discriminant validity among latent variables was evaluated using the Heterotrait–Monotrait Ratio (HTMT) [58]. The results are presented in Table 5.

**Table 5:** Heterotrait-Monotrait Ratio (HTMT) for Discriminant Validity

Model	Construct Pair	HTMT	95% CI (Bootstrap)	Threshold (0.85)
Model 1	ASÖ ↔ Mental Distress	0.694	[0.65–0.73]	< 0.85
Model 2	ASÖ ↔ GAD-7	0.717	[0.68–0.74]	< 0.85
Model 2	GAD-7 ↔ Depression	0.834	[0.81–0.86]	< 0.90
Model 2	GAD-7 ↔ Anxiety	0.824	[0.80–0.85]	< 0.90
Model 2	Depression ↔ Anxiety	0.878	[0.85–0.89]	< 0.90

For Model 1, the HTMT value between the Academic Stress Scale (ASÖ) and Mental Distress, specified as a second-order factor, was 0.694 (95% CI: 0.65–0.73), which is below the conservative threshold of 0.85.

In Model 2, the HTMT value between ASÖ and GAD-7 was 0.717 (95% CI: 0.68–0.74), also below the 0.85 threshold.

Among the psychological distress constructs in Model 2, the HTMT values were 0.834 for GAD-7 and Depression (95% CI: 0.81–0.86), 0.824 for GAD-7 and Anxiety (95% CI: 0.80–0.85), and 0.878 for Depression and Anxiety (95% CI: 0.85–0.89). All confidence intervals remained below 1.00.

## Discussion

### Overview

This study tested and compared two competing SEM specifications of the pathways from sociodemographic and lifestyle predictors to psychological distress in dental students, with academic stress as the central mediating variable. Both the transdiagnostic Mental Distress model (Model 1) and the GAD-7 specific anxiety model (Model 2) demonstrated strong mediation effects and acceptable-to-excellent fit. Model 1 offered superior global fit across all indices and is therefore recommended as the primary theoretical representation; however, Model 2 offers complementary clinical benefit by highlighting the role of clinically recognizable anxiety symptomatology as defined by GAD-7.

### Strengths of the study

Key strengths include the simultaneous testing of two theoretically distinct mediation specifications within the same sample, the use of latent variable modelling with bootstrap estimation, and the integration of both transdiagnostic and clinically actionable mediators. To our knowledge, this is the first study in the dental education literature to formally compare these competing SEM approaches.

### Convergence across both specifications

The two models were remarkably consistent in their substantive conclusions. In both, academic stress functioned as a potent full mediator, transmitting the combined influence of economic hardship, gender, academic year, and sleep onto psychological distress outcomes. This convergence strengthens confidence in the robustness of these pathways: they are not model-specific artefacts but reflect genuine structural relationships in the data. The dominant role of economic difficulties (β > 0.25 in both models) is consistent with research identifying financial strain as a primary driver of student mental health in health-professional programs [22, 61]. The protective effect of sleep duration (β ≈ −0.13 to − 0.15) also replicates robustly across specifications, reinforcing the importance of sleep as a modifiable determinant of mental health [25, 62].

### The transdiagnostic mental distress model (Model 1)

The transdiagnostic framing of the primary model reflects contemporary psychopathology theory, which emphasizes shared variance between internalizing disorders particularly anxiety and depression rather than treating them as fully independent constructs [63, 64]. The extremely high loading of academic stress onto the Mental Distress latent factor (β = 0.867) suggests that, in dental education, academic stressors are among the most potent single determinants of the overall psychological distress burden. The factor loadings for both DASS-21 Depression (β = 0.940) and DASS-21 Anxiety (β = 0.961) onto the Mental Distress construct confirm that distress, as experienced by dental students, is primarily transdiagnostic: anxiety and depression symptoms co-vary strongly, consistent with the Alqarni et al. [65] unified treatment conceptualization.

Model 1 also achieved the most parsimonious fit with fewer estimated parameters (55 vs. 67) and substantially lower information criteria (AIC = 461 vs. 781; BIC = 665 vs. 1029), indicating that the latent Mental Distress structure provides a more efficient representation of the covariance structure.

### The GAD-7 anxiety-specific model (Model 2)

Model 2 demonstrated that the clinically familiar GAD-7 performs well as a structural mediator, with academic stress explaining 78% of variance in the GAD-7 latent factor. Although slightly inferior to Model 1 on fit indices, Model 2 conveys a practically important message: the anxiety symptom profile captured by the GAD-7 is itself a powerful intermediate stage on the pathway from educational stressors to depression. This finding has direct clinical utility. The GAD-7 is already routinely available in clinical practice worldwide and has established diagnostic cut-points (mild ≥ 5, moderate ≥ 10, severe ≥ 15) that are interpretable by clinicians, counsellors, and student health teams without specialist psychometric training [19]. Our results suggest that deploying the GAD-7 as a screening and monitoring tool within dental schools could serve as an early-warning indicator for students at risk of developing depressive episodes, a secondary prevention strategy consistent with stepped-care models [66].

Every unit increase in academic stress score translated into a meaningful increase in GAD-7 scores (β = 0.884). Accordingly, targeting the drivers of academic stress is not only beneficial for general wellbeing but is also quantifiably linked to reduced anxiety symptom burden as measured by one of the most widely used clinical screening tools globally.

### Discriminant validity and transdiagnostic interpretation

The HTMT findings provide important insights into the relationships among the latent constructs. While discriminant validity was clearly supported between academic stress (ASÖ) and psychological distress variables (HTMT < 0.85), relatively higher HTMT values were observed among the psychological distress constructs themselves.

Given the conceptual relatedness of anxiety and depression, the use of the more liberal 0.90 threshold is appropriate [58]. All HTMT values remained below this threshold (GAD‑7 ↔ Depression = 0.834; GAD‑7 ↔ Anxiety = 0.824; Depression ↔ Anxiety = 0.878), indicating that these constructs, despite their strong associations, remain empirically distinguishable.

The elevated HTMT values, particularly between depression and anxiety (HTMT = 0.878), are theoretically expected and consistent with prior literature documenting their substantial overlap [17, 67]. This pattern reflects the shared variance between internalizing symptoms and supports the notion of a broader underlying liability (the p‑factor).

Importantly, these findings lend support to the transdiagnostic perspective adopted in this study. The strong interrelationships among anxiety- and depression-related constructs suggest that modelling them as manifestations of a higher-order Mental Distress factor (Model 1) provides a parsimonious and theoretically coherent representation.

Nevertheless, Model 2 offers distinct clinical utility. While Model 1 provides a more parsimonious higher-order structure, Model 2 employs the GAD-7, a well-validated clinical screening instrument widely used in primary care and mental health settings. The HTMT values for GAD‑7 with Depression (0.834) and Anxiety (0.824) remained below the 0.90 threshold, confirming that GAD-7, despite its conceptual overlap with these constructs, captures a distinct aspect of the internalizing spectrum. This supports the use of GAD-7 as a clinically actionable mediator that can be readily administered and interpreted in real-world settings, where a transdiagnostic latent variable may be less directly applicable.

Thus, rather than indicating a limitation, the observed pattern of associations strengthens the justification for the proposed higher-order structure (Model 1) for theoretical parsimony, while simultaneously validating the clinical utility of GAD-7 (Model 2) as a practicable screening tool for interventions targeting academic stress.

### Sociodemographic and lifestyle predictors

The present findings replicate and extend prior work. Economic difficulties emerged as the strongest single predictor of academic stress across both models, consistent with reports that financial constraints amplify the perceived demands of dental education particularly in programs requiring costly clinical equipment and laboratory materials [4, 68]. Institutions should consider financial support mechanisms (bursaries, reduced material costs, flexible payment schemes) as mental health interventions, not merely social welfare provisions.

The gender effect (β ≈ 0.21; female > male) is consistent with the broader literature on anxiety vulnerability [24] and may reflect intersecting factors including perfectionism, societal expectations regarding female emotional expression, and differential use of maladaptive coping strategies [6, 69]. The academic year effect (β ≈ 0.22; higher year = more stress) likely captures the accumulating burden of clinical exposure as students’ progress into direct patient contact, a pattern documented in longitudinal work on dental student burnout [21, 70].

Sleep duration was the only modifiable lifestyle variable examined and showed a consistent protective relationship (β ≈ −0.13 to − 0.15). Mechanistically, sleep supports hippocampal memory consolidation, prefrontal regulatory control of affect, and hypothalamic-pituitary-adrenal axis normalization, all of which reduce subjective stress appraisal [71]. Sleep hygiene programs have demonstrated efficacy in reducing anxiety and burnout in medical students [72] and represent a low-cost, high-reach intervention modality suitable for dental school wellness programs.

### Clinical and educational implications

The convergent evidence from both model specifications supports a multi-level intervention framework for dental schools. At the institutional level, curriculum streamlining, better support during the preclinical-to-clinical transition, and financial relief measures should be prioritized to reduce the primary source of stress. At the individual level, early screening using the GAD-7 is recommended: as Model 2 demonstrates, GAD-7 scores are structurally linked to both antecedent academic stressors and subsequent depressive outcomes, making them a practical sentinel indicator. Students scoring ≥ 10 on the GAD-7 (moderate anxiety) merit prompt referral to psychological support services. At the individual level, routine GAD-7 screening is recommended as a practical, low-burden tool for early identification of at-risk students. Combining these approaches may interrupt the stress → anxiety → depression pathway documented in both models.

### Limitations and future directions

Several limitations constrain the interpretability of the present findings. The cross-sectional design precludes causal inference; prospective or longitudinal studies are needed to establish temporal ordering among predictors, mediators, and outcomes. The single-institution sample from Turkey limits generalizability to other cultural contexts or dental education systems, although the consistency of findings with international literature is reassuring. Self-report measures are susceptible to response bias; biological or observer-rated measures of stress and distress would strengthen future replications. Important moderators including coping style, social support, personality traits such as perfectionism and conscientiousness, and sense of coherence were not assessed and may explain residual variance in distress outcomes [39, 73].

Several methodological limitations warrant consideration. First, treating academic year as a continuous predictor assumes linear increases in stress across training stages, an assumption that may not hold given the discrete nature of the preclinical-to-clinical transition. Second, the competing model specifications were compared using information criteria (AIC, BIC) rather than a formal chi-square difference test, as the two models are not nested; researchers should be aware that non-nested model comparisons carry inherent uncertainty.

Future research should prioritize longitudinal designs capable of establishing directionality, particularly examining whether anxiety (as captured by the GAD-7) prospectively predicts depressive episodes in dental students. Moderated mediation analyses exploring whether the academic stress pathway differs as a function of coping resources or social support would further enrich the model. Finally, randomized controlled trials of targeted interventions combining financial relief, sleep hygiene programs, and GAD-7-guided early referral are needed to confirm whether attenuating the identified stress pathways translates into meaningful reductions in clinically significant anxiety and depression.

## Conclusions

This study provides robust evidence that academic stress functions as the central mediating pathway through which economic difficulties, gender, academic year, and sleep duration exert their combined influence on psychological distress in dental students. Two competing SEM specifications converged on this conclusion: the transdiagnostic Mental Distress model demonstrated superior parsimony and fitness, while the GAD-7 model highlighted the specific role of clinically recognizable anxiety symptomatology. Both models confirmed full, bootstrap-validated mediation for all predictors. The GAD-7, as a globally recognized screening instrument, was shown to capture a mediating anxiety construct that is directly linked to both upstream academic stressors and downstream depressive outcomes strengthening the case for its routine deployment in dental school welfare programs. Interventions addressing economic hardship and sleep quality, combined with early GAD-7 screening and stepped-care referral, represent an evidence-informed approach to safeguarding mental health in dental education. Such evidence-based efforts have the potential to meaningfully improve the mental health and wellbeing of future dental professionals.

## Implications

### Theoretical implications

This study contributes to the growing body of evidence supporting a mediation framework in which academic stress operates as the primary pathway through which contextual and demographic factors translate into psychological distress among dental students. By testing two competing structural models and confirming the robustness of the mediation structure across both, the study strengthens the theoretical case for academic stress as a central explanatory construct in dental student wellbeing research. The integration of GAD-7 indicators as a competing measurement approach also advances methodological discourse on how anxiety constructs are best operationalized in this population.

A key theoretical contribution is the demonstration that a transdiagnostic Mental Distress model (Model 1) provides a superior model fit compared to an anxiety-specific GAD-7 model (Model 2). This finding supports the conceptualization of depression and anxiety as manifestations of a shared internalizing distress dimension in dental student populations, consistent with the tripartite model [17]. Future research should adopt transdiagnostic frameworks when investigating psychological distress in health professions education.

### Practical and clinical implications

The findings carry several actionable implications for dental school administrators, educators, and mental health providers.

Institutional Level: Economic status emerged as the strongest predictor of academic stress (β = 0.269), suggesting that financial support mechanisms including scholarships, emergency funds, and reduced fee structures may be among the most impactful institutional interventions. Dental schools should consider these not merely as financial aid but as mental health interventions.

Program Level: Gender differences in academic stress exposure (β = 0.212) highlight the need for gender-sensitive support programs within dental faculties. The protective effect of sleep duration (β = −0.131) points to the importance of workload management and schedule design that allow adequate recovery time, particularly during the preclinical-to-clinical transition period.

Clinical Screening: The GAD-7 model (Model 2) demonstrated that clinically recognizable anxiety symptoms serve as a powerful mediator between academic stress and depression. Given that the GAD-7 is already widely used in clinical practice, routine deployment of this scale in dental schools is recommended as an early-warning screening tool. Students scoring ≥ 10 (moderate anxiety) should be promptly referred to psychological support services.

Intervention Targeting: The fact that academic stress fully mediates all relationships underscores the value of targeted stress management interventions as upstream levers for reducing depression and anxiety.

### Policy implications

Dental education policymakers should consider curriculum reforms that reduce modifiable stressors, particularly during the transition from preclinical to clinical training. Accreditation bodies may consider mandating student mental health support services and routine anxiety screening as standard requirements for dental program certification.

## Supplementary Information

Supplementary Material 1.

## Data Availability

The datasets generated and/or analyzed during the current study are not publicly available due to institutional data protection regulations but are available from the corresponding author on reasonable request.

## Abbreviations

AIC: Akaike Information Criterion
AGFI: Adjusted Goodness-of-Fit Index
AMOS: Analysis of Moment Structures
ASÖ: Academic Stress Scale (Turkish version)
AVE: Average Variance Extracted
BC CI: Bias-corrected bootstrap confidence interval
BIC: Bayesian Information Criterion
CFA: Confirmatory factor analysis
CFI: Comparative Fit Index
CMIN/DF: Chi-square divided by degrees of freedom
CR: Composite Reliability
DASS-21: Depression Anxiety Stress Scales-21
Dep: Depression
GAD-7: Generalized Anxiety Disorder-7
GFI: Goodness-of-Fit Index
NFI: Normed Fit Index
PCLOSE: p value for test of close fit
RMSEA: Root Mean Square Error of Approximation
R²: Coefficient of determination
SEM: Structural equation modelling
SRMR: Standardized Root Mean Square Residual
TLI: Tucker–Lewis Index
