# Supplementary material for: Multidimensional predictors of psychological distress in dental education: the mediating role of academic stress a structural equation modelling study with competing model specification
Source: BMC Med Educ. 2026 Jul 31;26:1734. doi: 10.1186/s12909-026-09957-3 (PMC13712016; doi:10.1186/s12909-026-09957-3)
Supplement: Supplementary file 1 [file 12909_2026_9957_MOESM1_ESM.docx]

**Multidimensional Predictors of Psychological Distress in Dental Education: The Mediating Role of Academic Stress**

Supplementary Table S1. Demographic and Lifestyle Variables

| Questions(Türkçe) | Questions(English) | Coding / Categories |
| --- | --- | --- |
| Cinsiyet | Gender | 0 = Male  1 = Female |
| Uyku süresi | Sleep Duration | 1 = < 4 hours 2 = 4–6 hours 3 = 6–8 hours 4 = > 8 hours |
| Ekonomik Zorluk | Economic Difficulties | 1 = No difficulty 2 = Slight difficulty 3 = Moderate difficulty 4 = High difficulty 5 = Severe difficulty |
| Akademik Yıl | Academic Year | Continuous variable (1–5) |

Supplementary Table S2. Perceived Stress Items (ASÖ Items)

| **Item No** | Questions(Türkçe) | Questions(English) |
| --- | --- | --- |
| ASÖ1 | Beklenmedik bir olay karşısında sıkıntı yaşadım | I was upset because of something that happened unexpectedly |
| ASÖ2 | Önemli şeyleri kontrol edemedim | I felt that I was unable to control important things in my life |
| ASÖ3 | Kendimi stresli hissettim | I felt stressed |
| ASÖ4 | Kontrolüm dışındaki olaylar beni öfkelendirdi | I felt angered because of things that were outside of my control |
| ASÖ5 | Üstesinden gelemediğim zorluklar yaşadım | I felt difficulties were piling up so high that I could not overcome them |
| ASÖ6* | Kişisel sorunlarımı çözme konusunda kendime güvendim | I felt confident about my ability to handle my personal problems |
| ASÖ7* | Yaşamımda her şeyin yolunda gittiğini hissettim | I felt that things were going my way |
| ASÖ8* | Öfkemi kontrol edebildim | I was able to control my irritations |
| * Reverse-coded items | | |

Supplementary Table S3. GAD-7 (Generalized Anxiety Disorder Scale) Items

| **Item No** | Questions(Türkçe) | Questions(English) |
| --- | --- | --- |
| GAD1 | Gergin, endişeli veya sinirli hissetme | Feeling nervous, anxious, or on edge |
| GAD2 | Endişeyi durduramama veya kontrol edememe | Not being able to stop or control worrying |
| GAD3 | Çok fazla veya farklı konularda aşırı endişe | Worrying too much about different things |
| GAD4 | Rahatlamakta zorlanma | Trouble relaxing |
| GAD5 | Huzursuzluk nedeniyle yerinde duramama | Being so restless that it is hard to sit still |
| GAD6 | Kolayca sinirlenme veya çabuk öfkelenme | Becoming easily annoyed or irritable |
| GAD7 | Kötü bir şey olacakmış gibi korku hissetme | Feeling afraid as if something awful might happen |

Supplementary Table S4. DAS21 Depression Items

| Item No | Questions(Türkçe) | Questions(English) |
| --- | --- | --- |
| DEP1 | Olumlu duygular hissedemeyecekmişim gibi geldi | I felt that I could not experience positive feelings at all |
| DEP2 | Bir şeylere başlamakta zorlandım | I found it difficult to initiate activities |
| DEP3 | Hiçbir beklentim olmadığını hissettim | I felt that I had nothing to look forward to |
| DEP4 | Moralim bozuk ve keyifsizdim | I felt down-hearted and depressed |
| DEP5 | Hiçbir şeye karşı istek duyamadım | I was unable to become enthusiastic about anything |
| DEP6 | Kendimi değersiz hissettim | I felt that I was worthless |
| DEP7 | Hayatın anlamsız olduğunu hissettim | I felt that life was meaningless |

Supplementary Table S5. DAS21 Anxiety Items

| **Item No** | Questions(Türkçe) | Questions(English) |
| --- | --- | --- |
| ANX1 | Ağzımın kuruduğunu fark ettim | I was aware of dryness of my mouth |
| ANX2 | Egzersiz yapmadığım halde nefes alma güçlüğü yaşadım | I experienced breathing difficulty (e.g., excessively rapid breathing, breathlessness in the absence of physical exertion) |
| ANX3 | Titreme (örn. ellerde) hissettim | I experienced trembling (e.g., in the hands) |
| ANX4 | Kendimi aptal gibi hissedebileceğim durumlardan endişe duydum | I was worried about situations in which I might panic and make a fool of myself |
| ANX5 | Paniklemeye yakın olduğumu hissettim | I felt I was close to panic |
| ANX6 | Egzersiz yapmama rağmen kalp atışlarımı hissediyordum | I was aware of the action of my heart in the absence of physical exertion (e.g., sense of heart rate increase, heart missing a beat) |
| ANX7 | Ortada neden yokken korktuğumu hissettim | I felt scared without any good reason |
